# An integrated microfluidic device for the sorting of yeast cells using image processing

**DOI:** 10.1038/s41598-018-21833-9

**Published:** 2018-02-23

**Authors:** Bo Yang Yu, Caglar Elbuken, Chong Shen, Jan Paul Huissoon, Carolyn L. Ren

**Affiliations:** 1 Department of Mechanical Engineering, Massachusetts Institute of Technology, Cambridge, MA USA; 2 Bilkent University, National Nanotechnology Research Center, Ankara, Turkey; 3 Neverfrost Inc, Kitchener, ON Canada; 4 Department of Mechanical and Mechatronics Engineering, University of Waterloo, Waterloo, Ontario N2L 3G1 Canada

## Abstract

The process of detection and separation of yeast cells based on their morphological characteristics is critical to the understanding of cell division cycles, which is of vital importance to the understanding of some diseases such as cancer. The traditional process of manual detection is usually tedious and inconsistent. This paper presents a microfluidic device integrated with microvalves for fluid control for the sorting of yeast cells using image processing algorithms and confirmation based on their fluorescent tag. The proposed device is completely automated, low cost and easy to implement in an academic research setting. Design details of the integrated microfluidic system are highlighted in this paper, along with experimental validation. Real time cell sorting was demonstrated with a cell detection rate of 12 cells per minute.

## Introduction

Optical observations of yeast cell morphology is a common practice in several areas of microbiological studies, such as cell cycle modeling^1–5^ and aging studies^6^. One of the important steps when studying yeast cells involves the identification and isolation of yeast cells that are in the process of “budding”. However, most existing methods require manually observing and labelling each individual cell using a microscope, which is time-consuming and often inconsistent. Therefore, developing an automated device that can identify and isolate cells based on optical morphological observations is crucial to the systematic study of yeast cells. This work aims at demonstrating an engineering system capable of automating this task.

Microfluidics has recently been used in a variety of single cell analysis with great success. Compared to the traditional, operator-based manual cell handling and identification methods, microfluidic approaches offer numerous advantages that include reduced sample and reagent volumes, increased detection accuracy, higher repeatability, ease of automation and low cost^7–10^. Huang *et al*.^11^ demonstrated the viability of microfluidic based single-cell analysis, with a device that could manipulate and lyse single cells for quantification of the protein contents using fluorescence detection. Taniguchi *et al*.^12^ reported the possibility of analyzing protein and mRNA expression in *E. coli*, using a microscopic imaging microfluidic cytometry, although the system lacks the ability to sort and manipulate the cells. Falconnet *et al*.^13^ presented a high-throughput microfluidic imaging system capable of tracking single yeast cells over multiple generations, although their application is limited to immobilized cells. Hansen *et al*.^14^ demonstrated that microfluidic systems can be used to control and measure single yeast cells. Lee *et al*.^6^ used a microfluidic device to isolate and observe the process of aging in yeast cells. Haandbaek *et al*.^15^ used microfluidic impedance cytometry to associate cell impedance data with yeast cell morphological phenotypes. In addition, single cell manipulation has been demonstrated using droplet-based microfluidic devices^16^ and combined optical tweezer and microfluidic chip^17^. The previous work in this area have suggested that microfluidic technologies can be used to automate the process of identifying and manipulating individual yeast cells for cell cycle research.

A majority of current microfluidic cell sorters use external^18,19^ or integrated optics^20–22^ for detection. These optical devices usually rely on fluorescence^18,19^ or scattered light^22^ for detection and can be packaged in very small form factors using optical fibers. However, these optical devices often suffer from alignment issues and low signal-to-noise ratio, as well as requiring special equipment to operate^23^. Recently, several attempts have been made toward detection using microscopic imaging^24–26^. Although image based detection methods are usually limited by the bulkiness and low acquisition speed of cameras, there are many unique advantages: (1) the external equipment (microscope and camera) are standard equipment in most laboratories; (2) advancements in image processing algorithms can be utilized to enhance quality and obtain morphological or sub-cellular information accurately; (3) the detection algorithm can be modified easily for different cells/particles without redesigning the mechanical components, and (4) alignment is simple. In addition, no special modifications are required for the microscope and it can still be used for other purposes. For low-throughput applications involving challenging identification tasks, microscopic imaging detection remains a much more cost-effective, accurate, adaptive, and user-friendly method over traditional optical detection techniques. Therefore, it has been chosen as the detection method in this study.

There are several potential mechanisms for fluid control in microfluidic platforms: flow rate control via syringe pumps; pressure control via pressure systems; electrokinetic control through applied electrical fields to the fluids, and others^27–29^. Cell sorting and isolation requires fast response of flow control which limits the use of syringe pumps due to their long settling time. Electrical kinetic control is also of limited use for cell studies due to the risk of harming the cells with high electric fields. The indirect use of electro-kinetic control (such as electro-osmotic pumps)^30^ usually requires complicated designs and also suffers from potential surface defects and electrolysis effects. In principle, pressure control provides an almost instantaneous response and is therefore chosen for this study, and is achieved using microfabricated elastomeric microvalves.

The elastomeric microvalve, first introduced by Unger *et al*.^31^, consists of a pneumatically actuated polydimethylsiloxane (PDMS) membrane that is sandwiched between two channels. This type of valve has a response time of milliseconds, no leakage or dead volume, and is simple to fabricate. Elastomeric microvalves have been used in a variety of microfluidic applications including flow cytometry. For example, Ohnuki *et al*. reported a microfluidic device that uses the membrane structure of the microvalve to trap yeast cells for high-magnification microscopic imaging^32^. Fu *et al*. created a cell sorter with integrated micropumps using this technology, and were able to successfully sort live *E. coli* cells^33^. Fu *et al*.’s work demonstrated that elastomeric valves can be used to make fully integrated cell sorters with pumping, flow switching, and the potential for the addition of many different operations; the valves are also gentle enough to handle bioparticles. This type of microvalve is chosen in this study to build a sorting mechanism.

This study aims to develop an automated system capable of identifying cells with particular morphological characteristics using optical imaging and a classification algorithm, isolating cells of interest using a microfluidic chip with on-chip valves and then pump the isolated cells back to the observation window for additional analysis (such as identifying fluorescent markers). The design and experimentation are detailed in the following sections. Section two describes the design of the cell sorter system including the microfludic chip, actuation components and control algorithms. Section three documents a set of experiments for the validation of design parameters, and in section four the actual cell sorting process is evaluated. In the remainder of this section, background information relating the cell cycle phases is presented.

### Cell morphology classification

**Figure 1:**
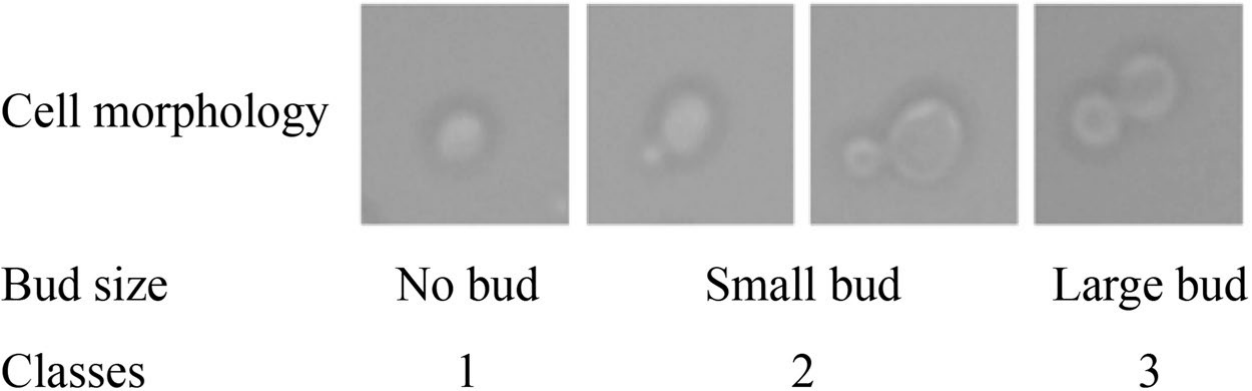
Yeast cell morphology through cell cycle progression. Small bud is defined to be less than ~50% of the mother cell in radius^34^.

Accurate identification of the cell cycle phase is vital to cell studies. An image processing and classification algorithm previously developed by our group^34^ is used in this study. This algorithm is capable of classifying microscopic images of yeast cells into three distinctive classes based on the existence and relative size of buds. Examples of each class and cell cycle phase are shown in Fig. 1.

The algorithm has three steps. The first step is the detection of a single yeast cell in an image. The second step is segmentation and feature extraction and the last step is classification. The classification portion uses a self-trained k^th^-nearest-neighbour (kNN)^35^ classifier (k = 3). The training data set consists of 30 samples in each class. The classifier has an average accuracy of 80%; details of the algorithm have been published previously^34^.

## Methods

### Sorting System Overview

The following design requirements were identified for the proposed cell sorter:

1. The cells must flow through the identification area one by one. The spacing between consecutive cells needs to be large enough to accommodate the delays associated with image processing and classification, as well as the response time of electro-mechanical components.
2. Cells in class 2 (as detected by the image processing algorithm) must be separated from other cells and collected.
3. The system must be able to pump the collected cells back through the identification area for additional analysis.
4. The microfluidic system must prevent cells from adhering to the channel walls.
5. The microfluidic system should be easy to fabricate with high reliability.

**Figure 2:**
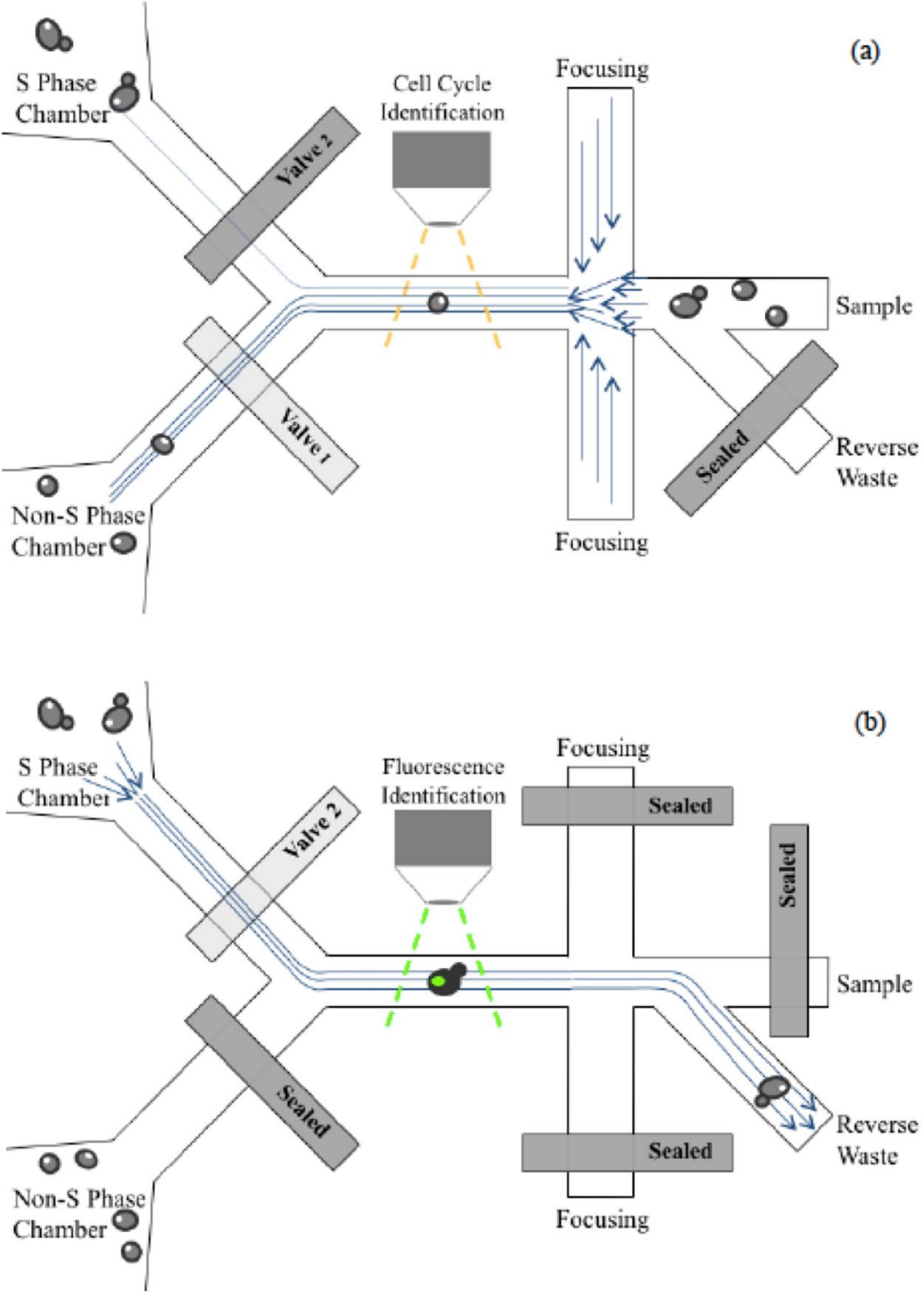
Schematics of the cell sorter: (**a**) in forward sorting mode the budding cells are identified by image processing and collected at one of the outlet chambers, (**b**) in reverse identification mode these collected cells are identified fluorescently.

Based on the above requirements, a cell sorter and its operating principle were proposed as illustrated in Fig. 2. It operates in two modes: the forward sorting mode and the reverse identification mode. In the forward sorting mode (Fig. 2a), the samples come in through the sample reservoir and pass through a flow focusing unit, which uses sheath flow to focus the sample stream and aligns the cells into a single line so that only one cell can pass through the cell identification region at a given time. This region is monitored by a Nikon Eclipse Ti microscope. A decision making algorithm processes the images to identify cells. If a cell is found, the system classifies it, and then opens/closes the two valves in the two sorting channels to direct the destination of the cell into either the collect reservoir (class 2 cells) or the waste reservoir (all the others). The forward sorting mode runs for a predefined period, during which the sorted cells are stored in a large chamber.

In the reverse identification mode (Fig. 2b), the budding cells stored in the chamber are pumped back towards a reverse waste reservoir (the sample and focusing reservoirs are now sealed), and these budding cells pass through the identification area again to look for the presence of the desired protein factor, which would be indicated by fluorescence markers.

### Experimental System

**Figure 3:**
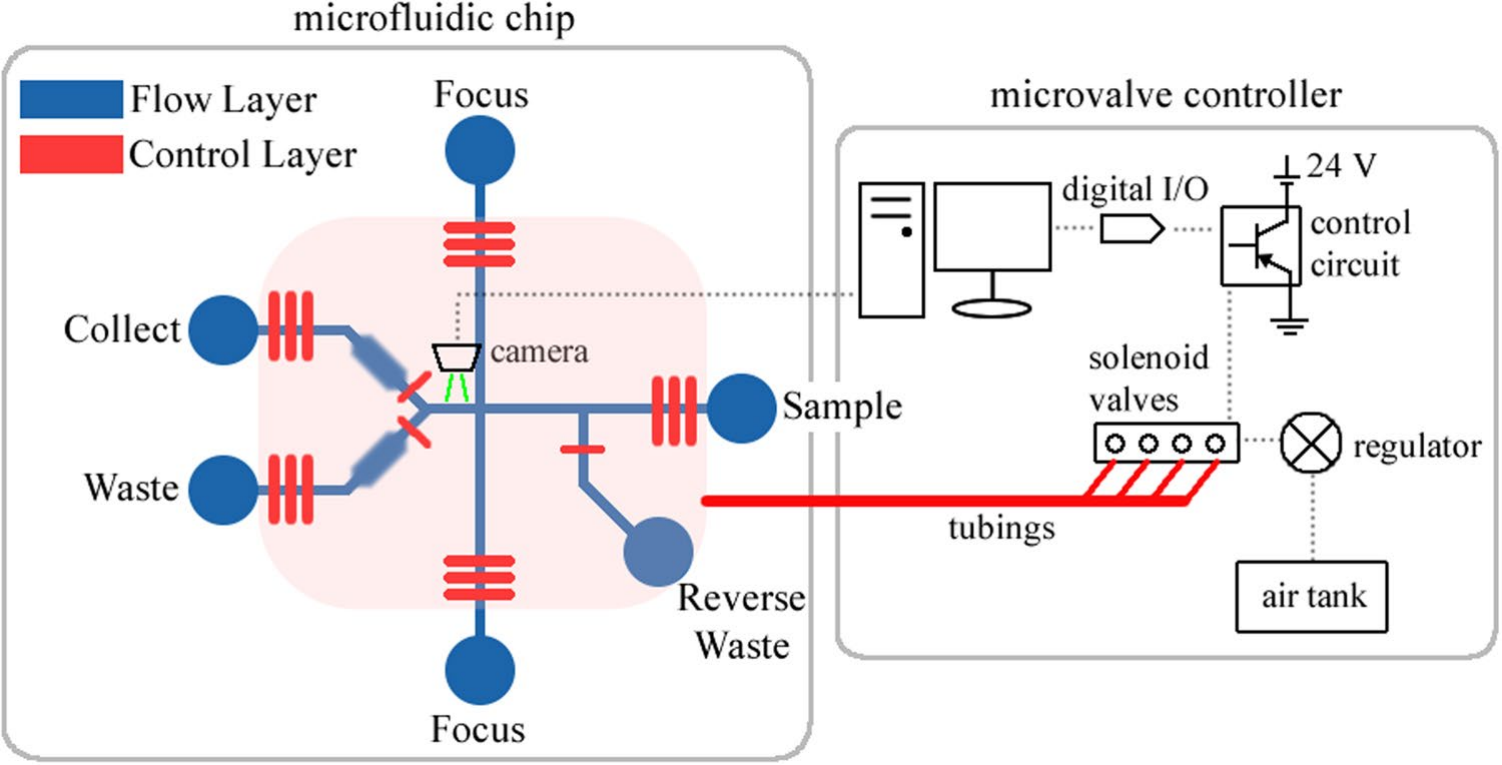
Schematic of the cell sorter device demonstrating the microfluidic chip and the microvalve controller.

The cell sorter system can be divided into two sub-systems: the microfluidic sorting chip and the electro-mechanical microvalve controller as shown in Fig. 3. The microchip, pumps and valves are part of the microfluidic sorting chip, while the microvalve controller is composed of the external components such as the camera, decision making unit, control circuitry, solenoid valves, regulator and air tank.

The microchip has two layers of channels. The top layer has the fluidic channels that consist of a sheath flow sample focusing structure, followed by a Y shape junction for sorting. The bottom layer has the control channels that control the integrated pumps and valves. The fluidic channels have a width of 60 μm and height of 20 μm. Since yeast cells are roughly 5~10 µm in diameter, a channel height of 20 μm was low enough to reduce out-of-plane movement of the cells while preventing cells from adhering to the channel walls. The control channels have widths of 100 μm, thus all valves have an active area of 60 × 100 μm. The chosen active area was a good balance between large volume displacement and reliability of the membrane. The peristaltic pumps are made of three valves in a row, with a distance of 100 μm between each valve. The control channels have a height of 40 μm and the membrane thickness is roughly 15 μm (1600 rpm PDMS spin coating). The control channel dimension and membrane thickness were chosen based on past experience that resulted in the most repeatable fabrication outcomes.

The sample channel and the two focusing channels are all 7.5 mm in length. Long microfluidic channels can help damp the pulsating effects of the peristaltic pumps. Equal-length channels ensure the pulses of the three pumps arrive at the focusing junction at the same time, to reduce mixing and backflow. The channel between the focusing junction and the sorting junction has a length of 1 mm; image capturing of the cell occurs in this part of the channel. Chambers 200 μm wide by 2 mm long are placed along the collect/waste channels. The reverse waste channel is placed half way between the sample reservoir and the focusing junction. A channel height of 20 μm also confines the cells (5~10 μm) to the center of the channel and thus 3D focusing was not needed.

Five sets of peristaltic pumps were included in the chip. Three were used to control the sample and the focusing flows for the forward mode; two pumps located downstream from the collect/waste chambers are used for the reverse mode. Three individual microvalves are used to control the reverse waste reservoir, and the collect and waste channels. Each individual microvalve is controlled using an external pneumatic valve. The timing of valve actuation is controlled with a micro-controller which takes higher level command from a PC running MATLAB via a serial port. Peristaltic pumping action is achieved with the switching sequence of: 110-011-001-101, where 0 and 1 indicate “valve open” and “valve close”, respectively. The time between each pattern in the sequence is called the pumping period, which can be varied between 10 ms to 500 ms. A delay can also be introduced between each sequence to alter the pumping speed (cycle delay) without changing the pumping period between pumps: 1 cycle delay between each pattern cycle is equivalent to half of the original speed, 3 cycles delay is quarter speed, and 7 cycles delay is one-eighth speed.

### Sorting Sub-System

**Figure 4:**
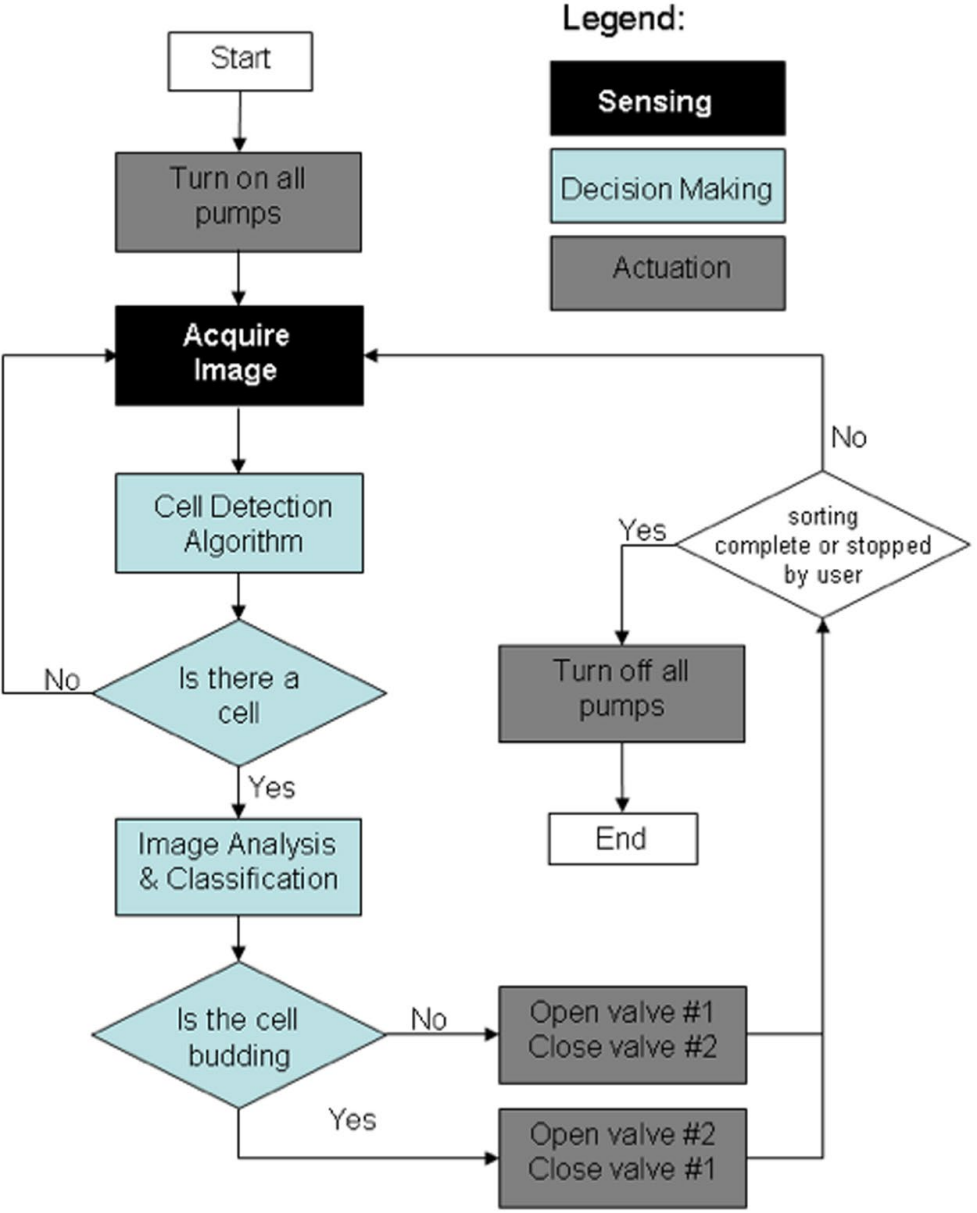
Flow chart of the sorting algorithm.

The sorting sub-system is responsible for sensing the yeast cells, making sorting decisions based on the observation, and then actuating the electro-mechanical components. The core functionality of the sorting sub-system is achieved through a software program. The flow chart in Fig. 4 illustrates the core functionality.

The sorting program was implemented in Matlab. The main loop of the program consists of the following actions:

1. Acquiring an image of the microfluidic channel.
2. Looking for a cell in the acquired image.
3. If a cell is found, performing image processing and classification on the cell.
4. Actuating sorting valves after classification.

Image acquisition was done through the use of the QImage CCD camera mounted on the microscope with a Matlab API provided by QImage. The magnification on the microscope was 30X (20X objective with a 1.5X internal multiplier), with a field of view of roughly 900 × 650 μm The QImage software allows the user to define a region of interest (ROI) so that only the part of the image that contains the channel is acquired, which reduces the acquisition time and also avoids the need to search non-channel areas. The captured image is first compared to a background image captured before the start of the sorting process to eliminate any artifacts attached to the channel. The cell detection algorithm scans the image from the upstream side toward the downstream side for the presence of a yeast cell. If a cell is not found, the image is discarded and the loop is repeated immediately.

If a cell is found, action items 3 and 4 are triggered. The geometrical features are extracted from the cell and the cell is classified. If the cell is classified as a Class 2, Valve 1 is closed and Valve 2 is opened so that the cell is propelled into the Collect Channel (Fig. 4); if the cell is not in Class 2, it is propelled through Valve 1 into the Waste Channel. After the cell has passed through, the valves are not switched until the next cell comes into the ROI and the valves are then only switched if the next cell is classified differently. While the program is running, all three pumps are on, and when the program is finished the pumps stop and seal the channels to prevent fluid flow due to the induced pressure differences. Different speed settings of the pumps result in different sorting speed and accuracy, the comparison of different settings are presented in section 3.

#### Sorting Mechanism Design

The ideal sorting mechanism for this system would be the following: placing the microscope camera ROI immediately upstream to the sorting junction, as the cell passes through the ROI, identify and classify the cell then immediately switch the direction of the flow to sort the cell into either the collect/waste channel. However, delays in the system make this very difficult to achieve.

The computing time of the image processing and classification algorithm is fairly predictable, however, the mechanical response time of the flow switching mechanism is unpredictable due to the limitation of the in-house PDMS chip fabrication process, which results in variations in the mechanical properties of PDMS. Due to the uncertainty in the delay, in this design the ROI is placed 500 μm upstream of the sorting junction. Observations showed that cells took 0.5~1 s to reach the sorting junction after passing through the ROI, which was enough time for flow switching to be completed.

In addition to the uncertainty in mechanical response time, the speed of the cell in the channel can also vary significantly due to the shape variation of the cell and having no control over its vertical location inside the channel. Predicting the actual speed of the cell would require upgrades to the existing optical hardware. Without knowing an individual cell’s speed inside the microchannel, it becomes very difficult to sort two cells that flow close to each other. This can be mitigated by diluting the cell concentration inside the microchannel to ensure proper spacing between consecutive cells. By doing so, the mechanical components of the sorting system are decoupled from the software: the software would only need to identify and classify cells as fast as possible, and turn on/off the corresponding valves whenever a cell is identified. The distance between the ROI and the sorting junction, as well as the large distance between consecutive cells would ensure all cells are properly sorted. while keeping the complexity of the system low.

#### Sorting Program Speed

The computation time required to complete one loop of the sorting program determines the maximum cell processing rate of the system. The CCD camera connected to the microscope takes full resolution (1600 × 1200 pixel) images at a frame rate of 5 frames per second (fps), although this can be increased to an effective 30 fps by defining a region of interest (ROI) no larger than 320,000 pixels. It can be assumed that image acquisition adds a fixed 33 ms processing time to the program.

The time required to run the cell detection algorithm is not constant. This algorithm works on the basis of scanning the pixels of the region of interest (ROI) in a predefined order until a ‘cell pixel’ is detected, whereupon the scan algorithm exits; thus an image frame without a cell takes longer to scan than a frame with a cell. Moreover, the size of the ROI also significantly affects the scan time. To reduce this further, only every fifth row in the ROI is scanned and along each row, only every fifth pixel is tested. The computation time of the detection algorithm was estimated to be approximately 4000 pixels/ms, obtained using the simulation described later in this section. An ROI of 600 × 170 pixels would require a maximum of 26 ms to scan completely.

The feature extraction and classification processes together require a consistent 35 ms to run. However, these processes do not run every program loop, but rather only when a cell has been detected in the scan algorithm. As mentioned before, the spacing between consecutive cells need to be large enough to ensure proper sorting. Assuming a cell is detected every 10 frames, the average time delay due to feature extraction and classification would be ~3.5 ms per loop. Combining these three sources of delay gave the total loop delay. The frame rate of the system (the number of frames it can scan every minute) is equal to the reciprocal of the total delay. The frame rate for different ROI sizes were tabulated in Table 1.

**Table 1:** Simulated Computational Delays for Different ROI Size.

Region of Interest [pixels (μm^2^)]		Max Scan Time (ms)	Worst-case Delay (ms)	Average Loop Delay (ms)	Average Frame Rate (fps)
1200 × 170	(440 × 60)	50	118	87	11.5
1000 × 170	(370 × 60)	42	110	79	12.6
600 × 170	(220 × 60)	26	94	63	15.8
340 × 170	(120 × 60)	15	83	52	19.2
170 × 170	(60 × 60)	8	76	45	22.2

Simulations were done to estimate the frame rates for different ROI sizes. The simulation applied the cell detection algorithm to a user generated image with no cells and used the Matlab timer function to obtain the time required to process this image. Five different ROI sizes were simulated: all have the same width of 170 pixels, since the micro channel width was constant and all were less than 640 × 480 pixels to ensure the maximum camera frame rate. The “Total Loop Delay” is obtained by adding 37 ms (33 ms image acquisition + 3.5 ms average classification) to the scan time to simulate the effect of image acquisition and feature extraction/classification.

The simulations showed that larger ROI sizes yield lower reduction in frame rate, as long as the maximum camera frame rate criterion is maintained. A large ROI could also result in one cell staying in the ROI over many frames and this cannot be accommodated with the current sorting system and algorithm design. A smaller ROI is therefore desirable, as long as no cells are missed. Successful operation is thus an inter-related combination of ROI, cell concentration, pumping speed, and computation time.

### Micro Fabrication

The chip was fabricated using standard multi-layer soft lithography techniques. The control and fluidic layers were cast from two different molds fabricated on silicon wafers. Both molds were made using photolithography, with the fluidic layer mold made of AZ50XT photoresist, while the control layer made of SU-8 photoresist. The microchip itself was cast using PDMS elastomer (Sylgard 184, Dow Corning).

To prepare the fluidic layer mold with semi-circular channels, a 4-inch silicon wafer was first dehydrated on an 115 °C hotplate for 20 min and cooled to room temperature. Hexamethyldisilazane (HMDS) was then spin-coated on the wafer to serve as an adhesion promoter for the AZ50XT photoresist. Next, AZ50XT photoresist was spin-coated on the silicon wafer at room temperature to form a 20 μm thick layer. The wafer was then baked on an 85 °C hotplate for 2 min, followed by 115 °C for 4 min, then let cool back to room temperature gradually. The photoresist was then UV exposed with a mask at 2500 mJ/cm^2^, and developed in AZ400K developer (1:3 developer/ultrapure water) for 4+ min. The wafer was then silanized in a vacuum desiccator along with a few drops of Tridecafluoro-1,1,2,2-tetrahydroctyl Triethoxy Silane for 2~3 h. Finally, the wafer is gradually heated to 125 °C (steps of 50 °C, 80 °C, 100 °C at 10 minute intervals) and baked for 30 min, then cooled down in two steps (80 °C and off). This heating process softens the photoresist and forms rounded features.

To prepare the control layer mold, a 4-inch silicon wafer was first dehydrated. A 5 μm thick adhesion layer was first spin coated using SU-8 2005, soft baked at 65 °C for 1 min, 95 °C for 2 min, 65 °C for 30 s and cooled down to room temperature. The wafer was then exposed without a mask at 250 mJ/cm^2^, then post baked at 65 °C for 1 min, 95 °C for 3 min, 65 °C for 30 s and cooled down to room temperature. Next, SU-8 2025 was spin-coated on top of the adhesion layer to form a 40 μm thick layer, soft baked at 65 °C for 3 min, 95 °C for 6 min, 65 °C for 30 s, and cooled down to room temperature. The wafer was then exposed using a negative mask at 630 mJ/cm^2^, and post baked at 65 °C for 1 min, 95 °C for 6 min, 65 °C for 30 s, 50 °C for 30 s, and rest on a glass surface for 2~3 h to reach equilibrium at room temperature. The wafer was then submerged in SU-8 developer for 5 min, then rinsed with fresh developer and water alternately until surface was free of impurities. Silanization was not necessary but recommended with the same procedure as described above.

**Figure 5:**
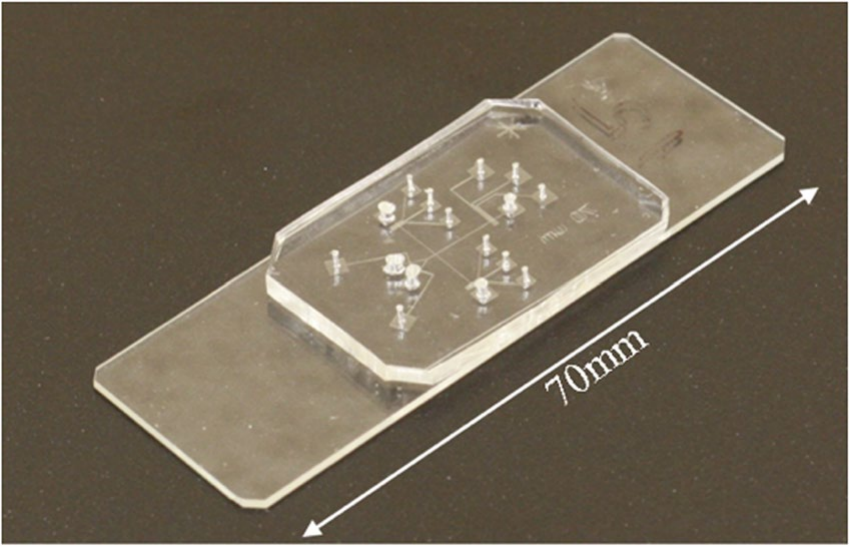
Final product of the flow cytometry microchip.

To create a PDMS chip, 50 g of 5:1 PDMS was mixed and degassed. Then ¾ of the mixture was poured onto the fluidic layer mold to form a 3–4 mm thick layer, which was then placed on an 80 °C hotplate for 10~12 min to let the PDMS cure slightly until just harden. The mold and replica should be removed from hotplate and let cool down to room temperature. Once cooled, the PDMS replica should be peeled off from the mold and cut into the desired shape. Access holes should be punched serving as the fluidic channel reservoirs. Then, 10 g of 20:1 PDMS was mixed, degassed, and spin-coated onto the control layer mold to a thickness of 60 μm, which was allowed to rest for 30 min to smooth out its surface. The mold and replica was placed on an 80 °C hotplate for 16~18 min, then removed from the hotplate and bonded with the PDMS replica with the fluidic channels immediately. The two PDMS replicas adhered to each other without plasma treatment. Then the remaining ¼ of the 5:1 PDMS mixture was poured on the control layer mold, around the newly bonded fluidic layer, and baked on an 80 °C hotplate for another 2~3 h to complete the curing reaction. The PDMS can then be peeled off, cut into desired shape, punched with access holes to the control layer. Finally, plasma treatment was performed to bond the PDMS structure with a glass substrate resulting in the chip shown in Fig. 5.

### Flow focusing

The performance of the flow focusing unit was experimentally tested under a variety of pumping speeds. Desirable focusing performances should have a narrow focused stream width and minimal mixing between the sample and focusing fluid. Fluorescent dye was used for the visualization of the focusing.

**Figure 6:**
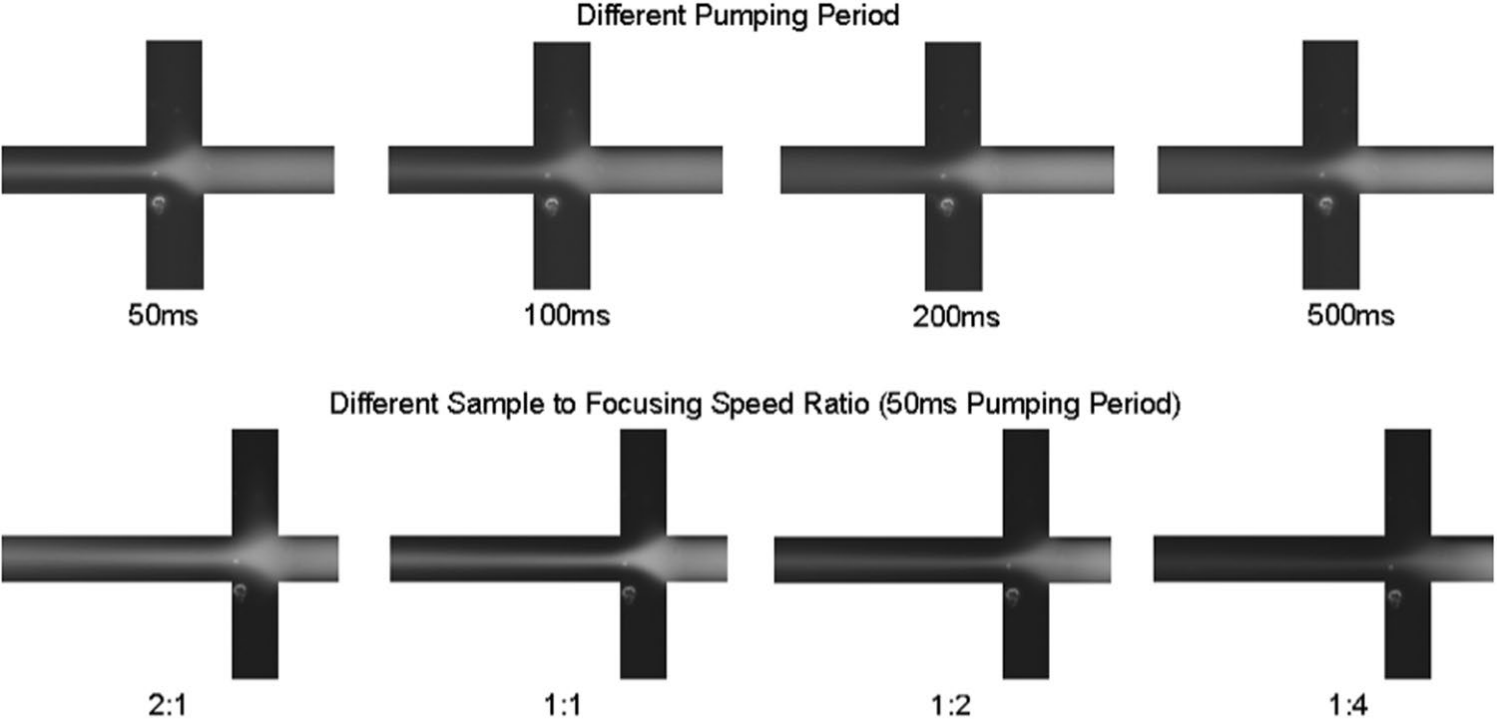
Flow focusing for different pumping period and different sample to focusing speed ratio.

The control channels for the pumps were first filled with water, then attached to the solenoid valves for pneumatic control. For the fluidic channels, all the reservoirs were filled with deionized water and pressurized until all the air bubbles were removed from the fluidic channel network, then the fluorescent dye solution was added to the sample reservoir and the pumps for both the sample and focusing channels were turned on. The pumps were allowed to operate until the fluorescent dye solution was completely propelled into the sample channel. The pumping period was varied between 50 ms and 500 ms (pumping frequency between 20 to 2 Hz) and cycle delays were added to either the sample channel pump or the focusing channel pump to produce uneven pumping speed. The images showing the focusing action for different conditions were shown in Fig. 6.

The results from different pumping periods showed that smaller pumping periods (higher pumping frequency) yielded a more confined focused stream with less diffusion across the streamlines. This was expected since with a high pumping frequency (above 20 Hz) the damping effect of the PDMS channel was more relevant, and thus the fluid velocity was smoother. With a lower pumping frequency, the pulsing effect from the peristaltic pump was stronger, resulting in stronger diffusion.

Different ratios of the flow speeds in the sample and focusing channels were created by adding cycle delays to the pumps driving the corresponding channels. For example, a sample to focusing speed ratio of 2:1 meant the sample channel pump was operated with no delay, while the focusing pumps had one cycle delay between each pattern cycle. Changing this ratio effectively changed the focusing stream width. Having a higher sample speed (ratio 2:1) than the focusing speed did not create a thin and focused stream as desired, while experimental observations show that a very low sample speed (ratio 1:4) resulted in unstable flow at the focusing junction: any disturbance could result in focusing fluid flowing back into the sample channel. In the result for the 1:2 speed ratio, the focused stream had the same width as the 1:1 ratio, but the brightness of the fluorescence was lower because of the lower concentration due to diffusion. Thus the 1:1 ratio was the best option. The focused stream width could be estimated by measuring the width of the bright stream in the images, and the stream width of the 1:1 ratio was estimated to be 15 μm. Since the yeast cells had diameters between 5 to 10 μm, this focused stream width was adequate for focusing yeast cells.

The flow focusing experiments showed that focusing with the micro fabricated peristaltic pump was difficult due to active mixing and unstable flow field, but it was possible to achieve good performance under optimized operating conditions. The pumps for all sample/focusing channels must operate at the same frequency and be synchronized to reduce active mixing. High pumping frequency would also be recommended.

### Sorting simulation using fluorescent beads

Before live yeast cells were tested in the chip, the sorting system was tested using fluorescent particles. The goal of this simulation was to determine the reliability and limitation of the sorting mechanism. Since the image analysis could not be applied to fluorescent particles, this simulation would only attempt to identify the particles passing through the region of interest (ROI), and then separate them based on their order of passing. For example, the 1^st^, 3^rd^, 5^th^… particles to pass through the ROI would be directed to one reservoir, while the 2^nd^, 4^th^, 6^th^… particles to go through would be directed to the other reservoir. The sorting program would need to be modified slightly. In the detection algorithm, the criterion for detecting a particle was now a threshold on the maximum pixel intensity in the ROI. The cell isolation, feature extraction, and cell classification algorithms were omitted. Since the particle detection algorithm was significantly different from the cell detection algorithm, the frame rate of the simulation was similar to but did not match the frame rates of the actual system.

**Figure 7:**
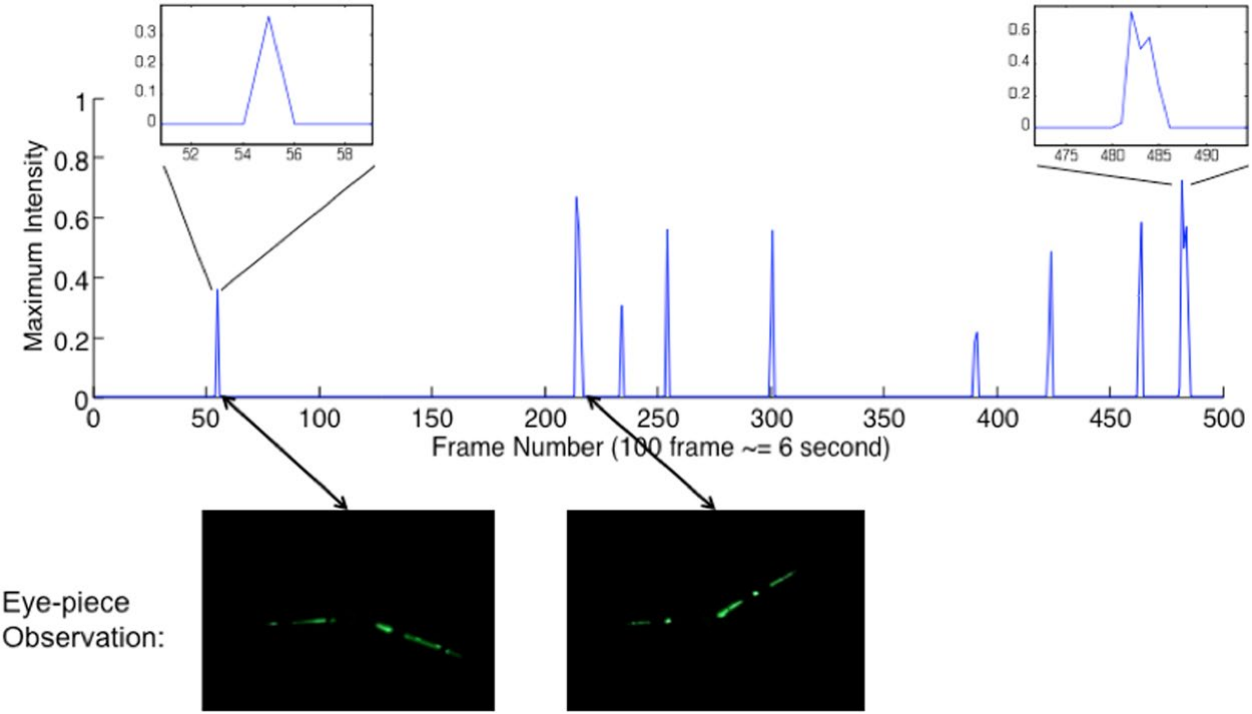
Sample plot of maximum intensity during sorting. The eye-piece observations show the path of a single fluorescent particle captured by the point-and-shoot camera.

The simulation was completed with a pumping frequency of 20 Hz (period 50 ms) and maintaining the same speed for all the pumps. The chip was filled with 1% Polyethylene glycol Diacrylate (PEGDA) aqueous solution, and pressures were applied to the inlets to remove any air bubbles trapped in the channels/reservoirs. The fluorescent particle solution was diluted to 0.1%, (about 2 × 10^7^ particles/ml), and 1% PEGDA was added. 10 µl of the fluorescent particle solution was added into the sample reservoir using a pipette, and the modified sorting program was started with the centre of the ROI set to approximately 500 μm upstream of the sorting junction. For evaluation purposes, the maximum pixel intensity in the ROI at each frame was saved, and the sorting process was recorded as a movie through the eye-piece using a point-and-shoot digital camera. Experiments were conducted for three different ROI sizes (μm × μm): 220 × 60, 120 × 60 and 60 × 60. 1500 video frames for experiment were recorded, which include approximately 30 to 40 particles being sorted. Then the recorded sorting movie was compared with the plot of maximum intensity during the sorting process; an example of such a plot is shown in Fig. 7.

Each peak in Fig. 7 corresponds to a fluorescent particle detected by the system, and the number of peaks should be equal to the number of particles observed through the eye-piece. The width of each peak represented the duration that a particle is visible in the region of interest, for example the first peak has a width of 1 frame, while the ninth peak has a width of 5 frames. Based on the number of frames in which a fluorescent particle is present, the speeds of the particles can be estimated. Observations showed that particle velocities are between 600 and 2000 μm/s.

By comparing the miss-sorting in the eye-piece observation and the peak plots, one could also determine the minimum inter-particle distance required for the sorting mechanism. Table 2 shows the tabulated simulation results. The results show that, for a region of interest equal to or smaller than 120 × 60 μm, the program could not guarantee that all the particles passing through are identified. This was consistent with the particle speed observation: a particle with a velocity of 2000 μm/s would pass through the 120 μm ROI in less than 60 ms, which is less than the 61 ms computing time for the simulation, while passing through the 220 μm ROI would take 110 ms. Referring back to Table 1 for the worst case loop time, assuming that yeast cells would have similar velocities to fluorescent particles, an ROI length of 220 μm would be large enough to accommodate the fastest particles/cells.

**Table 2:** Result of Fluorescent Particle Sorting Simulation.

Region of Interest (μm^2^)	Frame rate (fps)	Particles identified by program (%)	Minimum spacing between particles properly sorted (frames)
220 × 60	15.3	100%	6
120 × 60	16.4	90%	5
60 × 60	17.4	74%	4

The lower limit of the particle velocity suggested that, in the worst case, it would take approximately 800 ms for a cell to travel the 500 μm between the ROI and the sorting junction. This would be about 12 frames in the 16 fps system. Observations of the simulation suggested that if particles were within 6 frames of each other miss-sorting could occur. This is lower because most particles had velocities above the minimum observed velocity.

With the minimum spacing between the particles determined, and channel dimensions available, a limit on the cell concentration could be calculated using the formula:

1 $${C}_{sort}=1/(A\cdot {d}_{min})$$

where d_min_ is the minimum spacing between particles (500 μm), A is the cross-sectional area of the semi-circular micro channel, which can be approximated giving channel width (w) and height (h):

2 $$A=\frac{2}{3}\cdot w\cdot h$$

based on these formulae, with channel dimensions of 60 μm wide by 20 μm high, the maximum concentration of cells at the ROI is

3 $${C}_{sort}=2.5\times {10}^{6}\,{\rm{cells}}/{\rm{ml}}$$

However, the cell concentration at the ROI has been diluted by the sheath flow focusing. In addition, some cells will likely adhere to the bottom and walls of the microchip reservoir, thus the cell solution at the sample inlet should be at least 3 times more concentrated. Therefore, a safe cell concentration to ensure accurate sorting would be 1 × 10^7^ cells/mL.

## Results and Discussions

The experiments had validated all the necessary design components of the flow cytometry system. The design parameters are recapped in Table 3. An experiment was performed in the flow cytometry system to identify and sort yeast cells with small buds from the rest of the cells, using the reverse pumping mode for verification. The goal of this experiment was to verify the entire classification and sorting system including the reverse mode of the system.

**Table 3:** Design Parameters.

Chip design	Image system	Material
Fluidic channel dimensions: 60 μm wide by 20 μm high, sample/focusing channels length: 7.5 mm focusing junction to sorting junction distance: 1 mm collect/waste chamber: 200 μm × 2 mm Control channel dimensions: 100 μm wide by 40 μm high membrane thickness: 15 μm Valve/pump operation: pressure required: 160 kPa pumping period: 50 ms (20 Hz) all pumps maintain same speed	Nikon Eclipse Ti microscope, 20× objective with 1.5× internal multiplier. Region of interest (ROI): 600 × 170 pixel, or 220 × 60 μm^2^	Add 1% PEGDA in the cell culture media as surfactant; Use cell solution with a concentration between 0.5~1 × 10^7^ cells/mL

To prepare for the experiment, the control channels of the chip were filled with water and then connected to the pneumatic solenoid valves. The fluid channels were filled with cell culture media with 1% PEGDA, to ensure a safe and familiar environment for the cells and to reduce the effect of a rapidly changing environment. Meanwhile, the cell solution with a concentration of 1 × 10^7^ cells/mL was prepared, and kept agitated with a magnetic stirrer. The software was initialized to run for 300 loops in the forward mode, and then 300 loops in the reverse mode pumping back only the class 2 cells. The region of interest was set to an area approximately 500 μm upstream from the sorting junction to ensure there is enough time between the cell first captured on camera and sorted by switching the sorting valves to complete the classification and actuation actions. The program was slightly modified to save all the frames that contain cells, and the class that was assigned by the classifier. A pipette was used to deliver 10 μl of the cell solution into the sample reservoir, and then sorting was started.

**Figure 8:**
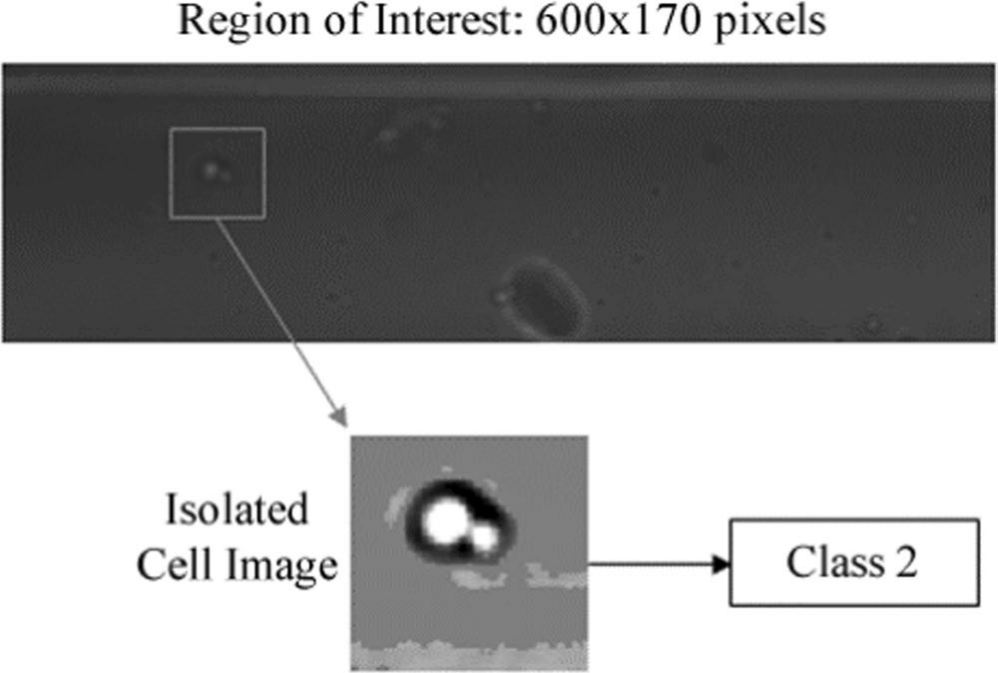
Example of image frame containing a cell.

The program was run 10 times for a total of 3000 loops to ensure an adequate number of cells were identified and sorted. In total, 37 cells were found; an example of a recorded image frame is shown in Fig. 8. 11 of the 37 were classified as Class 2, while in the reverse mode 12 cells were found.

### Classification Accuracy

The stored images of the detected cells were examined manually and their “true classes” were assigned. The confusion matrix for the classification result is shown in Table 4. Since the system was designed to sort class 2 cells from non-class 2 cells, the confusion matrix is also structured to show “class 2” and “not class 2” instead of showing three distinct classes.

**Table 4:** Confusion Matrix: live cell classification result.

N = 37		Classifier prediction:	
		Class 2	Not Class 2
Actual Class:	Class 2	9	6
	Not Class 2	2	20

For this system, the most critical measurement is the precision (positive predictive value) of the classifier. Precision is the ratio between the number of true positive predictions (number of actual class 2 cells that were also classified as class 2), and the number of predicted class 2 cells. The classification system should have high precision to ensure that the collected class 2 cells are indeed class 2 cells. The system demonstrated a precision of 9/11 = 81%, which is similar to the performance of the classifier demonstrated in our previous study^34^.

### Sorting Accuracy

The sorting results are shown in Table 5. As mentioned earlier, in the forward operation, class 2 cells were sorted to the collection reservoir while the non-class 2 cells were sorted to the waste reservoir, and in the reverse operation, only the cells in the collection reservoir were pumped back. One extra cell was found in the reverse mode, which was an error related to the sorting mechanism because two cells were too close together when being pumped through the ROI during the forward sorting.

**Table 5:** Sorting Performance of Flow Cytometry System with Live Yeast Cells.

	# of classification	
	Forward	Reverse (collect reservoir only)
Class 2	11	11
Not Class 2	26	1
Total	37	12

The experiment demonstrated that only one cell was sorted incorrectly out of 37 total cells. Assuming the probability of successful sorting is the same and independent for each cell, the sorting probability can be assumed to be a binomial distribution. Based on the experimental result, the confidence interval for the probability of successful sorting is [86.2%, 99.5%] (Wilson score interval, α = 0.05).

### Throughput

The average frame rate during the sorting process was 15 fps, and the observed cell detection rate was approximately 0.2 cells/s, which is the throughput of the cell sorting system. This detection rate was based on real-time analysis. The rate is significantly lower than the theoretical maximum throughput of 1.5 cells/s. The reason for the low throughput is primarily due to low flow rate inside the chip reservoir, therefore most of cells cannot be dragged into the channel to be detected. Future improvements to mitigate this issue include increasing the cell concentration, designing smaller reservoirs, surface treatment of the micro channel to reduce cell adhering, and adding an active cell agitation mechanism inside the reservoir. Other improvements to increase the system throughput include porting the sorting software onto a more powerful platform to increase frame rate, and fine tuning the mechanical system to make the flow switching response time shorter and more consistent. However some of these improvements may significantly increase the complexity and cost of the system.

## Conclusion

The sorting experiment shows that the entire cell sorter system is properly functional. It is capable of isolating class 2 cells in the forward sorting mode, and retrieving the stored cells in the reverse identification mode. The system is fully autonomous with consistent classification results, and can be used for the identification of protein factors immediately. This presented system provides a customizable platform for sorting of single cells based on image processing. Such a system would be a key technology for biologists, especially for synthetic biology studies which are attracting significant attention in the last few years. For these studies, researchers mostly use yeast cells to monitor GFP (green fluorescence protein) expression when studying genetically modified pathways. Sorting the GFP expressing cells from a large population can enable further gene editing for such a system.

This paper presented a microfluidic cell sorter integrated with on-chip microvalves and a classification and control system for the purpose of developing an automated research tool for classification and isolation of yeast cells in different cell cycle phases. The cell sorter uses microscopic image process for detection and integrated microvalves and pumps for fluid control. The operation of the sorting algorithm was highlighted, and the mechanical components, including sample focusing unit and the sorting mechanism, were thoroughly designed and validated through experiments. Finally, real time cell sorting was conducted to show the system is fully functional. It was determined that this system can accurately classify cells in the microfluidic environment, and can achieve a cell detection rate of 0.2 cells/s. The image processing algorithm can be readily modified to utilize the presented hardware for various other cell sorting applications.
